# *Burkholderia pseudomallei* in Unchlorinated Domestic Bore Water, Tropical Northern Australia

**DOI:** 10.3201/eid1707.100614

**Published:** 2011-07

**Authors:** Mark Mayo, Mirjam Kaestli, Glenda Harrington, Allen C. Cheng, Linda Ward, Danuta Karp, Peter Jolly, Daniel Godoy, Brian G. Spratt, Bart J. Currie

**Affiliations:** Author affiliations: Menzies School of Health Research, Darwin, Northern Territory, Australia (M. Mayo, M. Kaestli, G. Harrington, A.C. Cheng, L. Ward, B.J. Currie);; Royal Darwin Hospital, Darwin (L. Ward, B.J. Currie);; Northern Territory Department of Natural Resources, Environment and the Arts, Darwin (D. Karp, P. Jolly);; Imperial College, London, UK (D. Godoy, B.G. Spratt)

**Keywords:** Burkholderia pseudomallei, bacteria, melioidosis, bore water, Northern Australia, dispatch

## Abstract

To determine whether unchlorinated bore water in northern Australia contained *Burkholderia pseudomallei* organisms, we sampled 55 bores; 18 (33%) were culture positive. Multilocus sequence typing identified 15 sequence types. The *B. pseudomallei* sequence type from 1 water sample matched a clinical isolate from a resident with melioidosis on the same property.

*Burkholderia pseudomallei* is an environmental bacterium that causes melioidosis (*1*), a disease that is endemic throughout much of southeastern Asia and tropical northern Australia and sporadically occurs in other regions (*2*). Most infection is thought to result from percutaneous inoculation, but inhalation, aspiration, and ingestion of soil or water containing *B. pseudomallei* bacteria are the most recognized routes of infection. Outbreaks of melioidosis in Australia after exposure to contaminated water have been described. An outbreak of 159 cases in intensive piggeries (hog lots, a type of factory farm that specializes in raising pigs up to slaughter weight) in Queensland was attributed to contamination of the water supply (*3*), and a clonal outbreak in pigs on a small farm outside Darwin, Northern Territory was linked to *B. pseudomallei* cultured from the farm’s bore water (*4*). Two clonal clusters of human melioidosis have also been found in remote indigenous communities in northern Australia where molecular typing of recovered bacteria traced the source of infection to a contaminated community water supply. Fatalities occurred in both outbreaks. In 1 outbreak, the water supply was not chlorinated (*5*); in the other, the chlorination system was not adequately maintained (*6*).

Bore water can be contaminated with *B. pseudomallei* in our region (*4**,**7*). We surveyed a series of bores to ascertain how commonly such contamination occurs and whether *B. pseudomallei* is transient or persistent in positive bores. We then compared the genetic diversity of *B. pseudomallei* strains recovered from bores with strains from human melioidosis cases and other environmental strains from the region.

## The Study

Darwin, capital of the Northern Territory, Australia, is a coastal tropical city at 12°S. It has 2 distinct seasons: a hot monsoonal wet season from October through May and a dry season with very little, if any, rain from June through September. The city has a population of ≈100,000. Outside the city are many rural blocks of land 1–20 acres in size. Most have a family house, with cultivated gardens or native bush; domestic animals; and sometimes small numbers of farm animals, such as goats, pigs, and chickens. Horticultural activities include planting of mangoes, Asian vegetables, and watermelons. Most residents use unchlorinated groundwater provided by deep bores that tap into the underlying aquifers. We estimate that >3,000 such bores are in the rural areas and provide unchlorinated water to as many as 10,000 persons. Each year, 25–50 human cases of melioidosis occur in the Northern Territory; 50% occur in Darwin residents and 10%–15% occur in those living in rural areas surrounding Darwin (M. Mayo et al., unpub. data). Melioidosis also occurs in domestic and farm animals in the region.

We sampled bore water from 55 blocks in the Darwin rural region. All blocks were within a 30-km radius of Darwin, and all used unchlorinated bore water for domestic and irrigation purposes. Water samples were collected from the bore head (initial outlet of groundwater at source), water storage tank, and other water exit points (taps, hoses). For each sample, 1 liter of water was filtered through 0.22-µm filters (Millipore Corporation, Bedford, MA, USA). Filters were then cultured separately in Ashdown broth (Oxoid, Melbourne, Victoria, Australia) and tryptone soy broth (Oxoid) with gentamicin 10 mg/mL. Broth was plated onto Ashdown agar (Oxoid) on days 2, 7, and 14. Bacterial colonies suggestive of *B. pseudomallei* by morphologic appearance on Ashdowns agar were confirmed by Gram stain, oxidase test, agglutination with *B. pseudomallei* antiserum, and a specific PCR targeting *B. pseudomallei* type III secretion system (*8*). Confirmed *B. pseudomallei* bacteria were cultured on chocolate agar (Oxoid), and DNA was extracted by using a DNeasy tissue kit (QIAGEN, Hilden, Germany). Multilocus sequence typing (MLST) of bacterial DNA determined the sequence type (ST) for each isolate (*9*), allowing comparison with STs in the global MLST dataset (http://bpseudomallei.mlst.net).

*B. pseudomallei* was cultured from 18 (33%) of 55 water samples; 16 (36%) of 45 blocks tested during the wet season were positive, and 2 (20%) of 10 blocks tested during the dry season were positive. From 18 initial isolates, 9 distinct STs were identified; ST266 was found at 4 separate sites and ST109 at 3 (Table). Nine of the 18 positive sites were resampled 3 times during a 2-year period. In 5 (56%) of 9 sites, *B. pseudomallei* was recovered at least 1 additional time, and 3 sites were positive on 4 sampling occasions. STs of isolates from repeat sampling showed up to 3 different STs at the same location at the same time. At 1 site, the same ST (ST325) was present in all 4 samplings during the 2 years. Nevertheless, at each of the 5 sites with repeat positive cultures, including this site, an ST different from the original ST was recovered, even if the original ST was still present (Table).

**Table:** Sampling, culture, and MLST results from initial and repeat sampling of rural unchlorinated domestic water supplies, Northern Territory, Australia*

Site no.	1st sampling	2nd sampling	3rd sampling	4th sampling
1	109	Negative	Negative†	Negative
2	266	558, 326, 559	326, 559†	109
3	325	325	325, 328†	325†
4	109	Negative	334†	Negative
5	320	–	–	–
6	326	Negative	Negative†	Negative†
7	109	109	121†	109
8	132	–	–	–
9	325	Negative	Negative†	Negative
10	266	Negative	Negative†	Negative
11	266	–	–	–
12	330†	–	–	–
13	333†	333, 243†	Negative	Negative
14	132	–	–	–
15	266	–	–	–
16	132	–	–	–
17	109	–	–	–
18	131	–	–	–

*MLST, multilocus sequence typing; –, not resampled. †Indicates sampling during the dry season (June–September); 2nd–4th samplings were conducted during a 2-year period.

During the sampling period, a total of 15 distinct STs were recovered from water samples; of these, 10 were found in *B. pseudomallei* isolates collected from humans with melioidosis in the rural area (Figure), including 2 STs from fatal cases (ST109 and ST132). Of the 5 other STs, ST243 and ST334 occurred in humans in urban Darwin, and ST328 was recovered from a goat with fatal melioidosis. Although we do not have data on bacterial load in these positive water sources, the strain recovered from bore water at 1 location (ST131) was an identical ST to the *B. pseudomallei* isolate recovered from the sputum of a resident of that property who had nonfatal melioidosis pneumonia.

**Figure:**
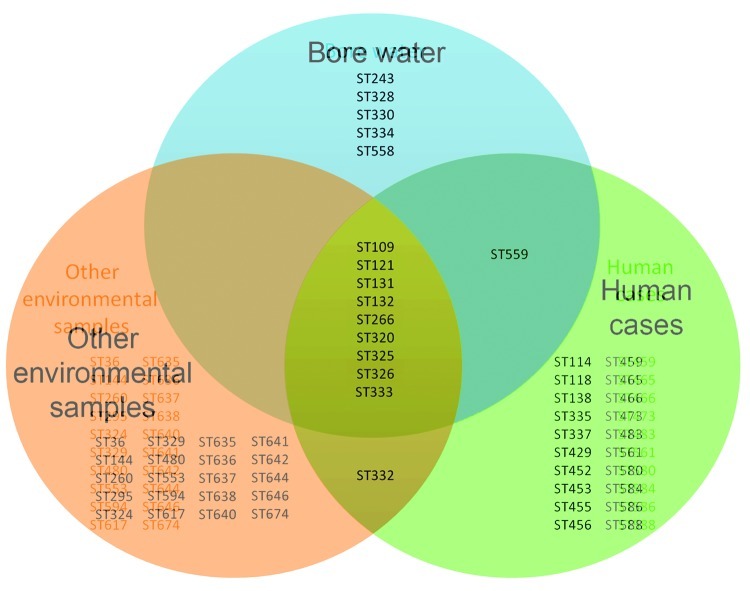
Venn diagram of sequence types (STs) determined by multilocus sequence typing found in *Burkholderia pseudomallei* strains from bore water (n = 15 STs), human cases (n = 31 STs), and other environmental samples (n = 30 STs) from the rural region of Darwin, Northern Territory, Australia.

Surveys of *B. pseudomallei* across northern Australia have shown a large genetic diversity among strains but distinct regional separations on MLST (*10*). Although the overall diversity of *B. pseudomallei* within Australia is considered greater than that seen in southeastern Asia (*11*), consistent with Australian *B. pseudomallei* lineages being ancestral to those elsewhere, environmental studies from Thailand have also shown enormous diversity in STs within a small geographic location (*12*). What remains unclear from these studies is whether differential virulence exists among environmental strains of *B. pseudomallei* and whether only a proportion of those isolates recovered from the environment have the potential to cause clinical disease (*13*).

Therefore, although STs found in this study were also represented in humans with melioidosis, the actual public health implications of the findings require further elucidation. Other variables require further investigation to determine the implications of our findings. These include bacterial load and differential bacterial virulence potential among the *B. pseudomallei* strains in water supplies. Additional considerations would be to quantify the infection risk potential from exposure to culture-positive water through ingestion or after aspiration or inhalation of droplets or aerosols containing *B. pseudomallei* during, for instance, showering.

## Conclusions

*B. pseudomallei* is common in unchlorinated domestic bore water supplies in the rural region of Darwin, Northern Territory, Australia. Initially, 33% of sites tested were positive for this bacterium, and more than half of these sites on at least 1 occasion were positive again when resampled. MLST showed a great diversity of STs, with persistence and variation in ST found on repeat sampling. STs often matched those found in humans with melioidosis from the same region. *B. pseudomallei* ST found in the sputum of 1 case-patient with melioidosis was a direct match to the ST of *B. pseudomallei* cultured from the bore water on the property on which this case-patient lived.

## References

[1] White NJ. Melioidosis. Lancet. 2003;361:1715–22. 10.1016/S0140-6736(03)13374-0 12767750

[2] Cheng AC, Currie BJ. Melioidosis: epidemiology, pathophysiology, and management. Clin Microbiol Rev. 2005;18:383–416. 10.1128/CMR.18.2.383-416.2005 15831829 PMC1082802

[3] Ketterer PJ, Webster WR, Shield J, Arthur RJ, Blackall PJ, Thomas AD. Melioidosis in intensive piggeries in south eastern Queensland. Aust Vet J. 1986;63:146–9. 10.1111/j.1751-0813.1986.tb02953.x 3753342

[4] Millan JM, Mayo M, Gal D, Janmaat A, Currie BJ. Clinical variation in melioidosis in pigs with clonal infection following possible environmental contamination from bore water. Vet J. 2007;174:200–2. 10.1016/j.tvjl.2006.05.006 16807011

[5] Currie BJ, Mayo M, Anstey NM, Donohoe P, Haase A, Kemp DJ. A cluster of melioidosis cases from an endemic region is clonal and is linked to the water supply using molecular typing of *Burkholderia pseudomallei* isolates. Am J Trop Med Hyg. 2001;65:177–9. 11561699 10.4269/ajtmh.2001.65.177

[6] Inglis TJ, Garrow SC, Henderson M, Clair A, Sampson J, O’Reilly L, *Burkholderia pseudomallei* traced to water treatment plant in Australia. Emerg Infect Dis. 2000;6:56–9. 10653571 10.3201/eid0601.000110 PMC2627980

[7] Inglis TJ, Foster NF, Gal D, Powell K, Mayo M, Norton R, Preliminary report on the northern Australian melioidosis environmental surveillance project. Epidemiol Infect. 2004;132:813–20. 10.1017/S0950268804002663 15473143 PMC2870167

[8] Novak RT, Glass MB, Gee JE, Gal D, Mayo MJ, Norton R, Development and evaluation of a real-time PCR assay targeting the type III secretion system of *Burkholderia pseudomallei.* J Clin Microbiol. 2006;44:85–90. 10.1128/JCM.44.1.85-90.2006 16390953 PMC1351940

[9] Godoy D, Randle G, Simpson AJ, Aanensen DM, Pitt TL, Kinoshita R, Multilocus sequence typing and evolutionary relationships among the causative agents of melioidosis and glanders, *Burkholderia pseudomallei* and *Burkholderia mallei.* J Clin Microbiol. 2003;41:2068–79. 10.1128/JCM.41.5.2068-2079.2003 12734250 PMC154742

[10] Cheng AC, Ward L, Godoy D, Norton R, Mayo M, Gal D, Genetic diversity of *Burkholderia pseudomallei* isolates in Australia. J Clin Microbiol. 2008;46:249–54. 10.1128/JCM.01725-07 18003806 PMC2224310

[11] Pearson T, Giffard P, Beckstrom-Sternberg S, Auerbach R, Hornstra H, Taunyok A, Phylogeographic reconstruction of a bacterial species with high levels of lateral gene transfer. BMC Biol. 2009;7:78. 10.1186/1741-7007-7-78 19922616 PMC2784454

[12] Wuthiekanun V, Limmathurotsakul D, Chantratita N, Feil EJ, Day NP, Peacock SJ. *Burkholderia pseudomallei* is genetically diverse in agricultural land in northeast Thailand. PLoS Negl Trop Dis. 2009;3:e496. 10.1371/journal.pntd.0000496 19652701 PMC2713400

[13] Currie BJ. Advances and remaining uncertainties in the epidemiology of *Burkholderia pseudomallei* and melioidosis. Trans R Soc Trop Med Hyg. 2008;102:225–7. 10.1016/j.trstmh.2007.11.005 18166205

